# BK Polyomavirus-associated nephropathy – diagnostic and treatment standard

**DOI:** 10.1093/ndt/gfaf002

**Published:** 2025-01-10

**Authors:** Mohammed Al-Talib, Matthew Welberry-Smith, Andrew Macdonald, Siân Griffin

**Affiliations:** Systems Immunity Research Institute, Division of Infection and Immunity, School of Medicine, Cardiff University, Cardiff, UK; Bristol Medical School, University of Bristol, 5 Tyndall Avenue, Bristol, UK; Faculty of Biological Sciences, University of Leeds, Woodhouse, Leeds, UK; Department of Renal Medicine and Transplantation, Leeds Teaching Hospitals NHS Trust, Beckett Street, Leeds, UK; Faculty of Biological Sciences, University of Leeds, Woodhouse, Leeds, UK; Department of Nephrology and Transplantation, Cardiff and Vale University Health Board, Cardiff, UK

**Keywords:** BK polyomavirus-associated nephropathy, diagnosis, pathophysiology, prognosis, treatment

## Abstract

BK polyomavirus (BKPyV) is recognized as a significant viral complication of kidney transplantation. Prompt immunosuppression reduction reduces early graft failure rates due to BK polyomavirus-associated nephropathy (BKPyVAN), however, modulation of immunosuppression can lead to acute rejection. Medium-to-long-term graft outcomes are negatively affected by BKPyVAN, probably due to a combination of virus-induced graft damage and host immune responses against graft alloantigens potentiated by immunosuppression reduction. Kidney biopsy remains the gold-standard diagnostic test, however, false-negative findings are common due to the focal nature of BKPyVAN. BKPyV DNAemia, as measured by quantitative polymerase chain reaction, is established as a screening test but there is at present no (inter)national standardization of these assays to allow collation and comparison of data between centres. Randomized controlled trials are lacking both in terms of optimal immunosuppression reduction strategies, and for the medications variably used to attempt treatment in clinical practice. Much of the fundamental biology of BKPyV is not yet understood, and further elucidation is required to promote rational direct-acting antiviral drug design. Insights into the role of adaptive immunity in control of BKPyV have informed the design of novel treatments such as adoptive immunotherapies and neutralizing antibodies that require evaluation in clinical studies. Here, we review the current standards of diagnosis and treatment of BKPyVAN and discuss novel developments in the pathophysiology, diagnosis, outcome prediction, and management.

IN A NUTSHELL

- Persistent, high-level BKPyV replication in kidney transplant recipients is associated with the development of BKPyVAN. BKPyVAN is associated with shortened graft survival.
- Histology remains the gold standard for the diagnosis of BKPyVAN. Sampling more than one core reduces the likelihood of a false-negative result. Thresholds of high-level or sustained DNAemia have been adopted in some screening strategies to define ‘presumptive’ BKPyVAN and guide proactive immunosuppression reduction prior to a drop in graft function.
- Immunosuppression reduction is the mainstay of management, however, high-quality evidence defining optimal strategies is lacking. A stepwise reduction in the burden of immunosuppression, beginning with either the antiproliferative agent or calcineurin inhibitor, is preferred. Multiple stepwise decreases may be required but must be balanced with the risk of precipitating acute rejection.
- No adjunctive treatment can be recommended in isolation due to lack of robust efficacy data. Intravenous immunoglobulin may be preferentially considered above other medications only after maximal acceptable reduction in immunosuppression. The lack of therapeutic options further emphasizes the need for better understanding of BKPyV biology and protective immunity to inform development of novel treatments.
- Elucidation of fundamental BKPyV biology is required to develop improved treatment options, including an understanding of viral cell entry, replication, and exit mechanisms (for antiviral targeting) and immune responses. Monitoring of BKPyV-specific cellular immunity may provide a route to guide individualized immunosuppression modification and prognostication of BKPyVAN.

## INTRODUCTION

BK polyomavirus (BKPyV) is a 40–45 nm non-enveloped, icosahedral, closed circular double stranded DNA virus of ∼5 kb, belonging to the same family of viruses as Merkel cell polyomavirus and JC polyomavirus. BKPyV is ubiquitous in humans, with seroprevalence rates in adults exceeding 90% [1, 2]. Genetic heterogeneity in the major capsid protein VP1 is used to classify BKPyV into four main subtypes, with genotype I being the most common (80%), followed by genotype IV (15%) [3, 4]. In immunocompetent individuals, BKPyV has no known clinical significance. Asymptomatic urinary shedding is common and suggests residence in the urinary tract [5]. Following kidney transplantation, immunosuppression may trigger viral replication as immune control is disrupted. Up to 30% of kidney transplant recipients (KTRs) will develop BKPyV DNAemia and, of those, approximately a quarter will develop BK polyomavirus-associated nephropathy (BKPyVAN) [6, 7]. These occur typically, although not exclusively, within the first year of transplantation when the immunosuppressive burden is at its highest [6]. Other manifestations of BKPyV replication post-transplantation include transplant ureteric stenosis, haemorrhagic cystitis (more common in allogeneic stem cell transplant recipients), and urothelial malignancy [8].

Numerous therapeutic interventions with promising *in vitro* activity against BKPyV lack clinical efficacy data. The mainstay of management is immunosuppression reduction, which is thought to facilitate viral clearance by allowing reconstitution of BKPyV-specific cellular immunity. However, this is coupled with the potential for triggering allograft rejection in both the short and longer term. Registry data from the UK, Australia, and New Zealand, indicate that BKPyVAN is the single cause for graft failure in <2% of recipients [9, 10]. However, BKPyVAN is associated with an increased risk of all-cause graft loss [10]. Prospective screening for BKPyV DNAemia post-transplantation allows proactive immunosuppression reduction prior to measurable graft dysfunction, although when considering any reduction in immunosuppression the immunological risk of the transplant should be taken into account. Advances in identifying patients at risk of progressing to BKPyVAN are needed to tailor immunosuppression reduction strategies and avoid exposing those at low risk to the risk of allograft rejection. Well-designed randomized controlled trials (RCTs) comparing immunosuppression reduction strategies, and assessing the efficacy of adjunctive therapies, are also a priority.

## DIAGNOSTIC STANDARDS AND NEW DEVELOPMENTS

A definitive diagnosis of proven BKPyVAN continues to rely on an allograft biopsy with characteristic cytopathic features and evidence of BKPyV infection (see Box 1 for consensus definitions). BKPyV appears to preferentially target renal tubular epithelial (RTE) and urothelial cells *in vivo*. In the context of immunosuppression following kidney transplantation, loss of immune control results in enhanced virus replication. Within RTEs, histological manifestations include nuclear enlargement and ground-glass intra-nuclear inclusions. These cells eventually detach and can be detected in the urine as ‘decoy cells’. Progression of BKPyVAN is associated with interstitial inflammation and tubulitis, with a prominent plasma cell component, and spread of involvement from distal to more proximal sections of the nephron. These features are non-specific, and BKPyVAN diagnosis is dependent on the concomitant identification of virus within the tissue. This is most commonly performed using immunohistochemical staining for the Simian Vacuolating Virus 40 (SV40) large T antigen (TAg), the antibody for which is cross-reactive against TAg expressed by BKPyV and other human polyomaviruses. This includes JC polyomavirus, which is typically associated with progressive multifocal leukoencephalopathy but can rarely cause a clinical and histological picture like BKPyVAN [11]. Less commonly used diagnostic options include *in situ* hybridization, quantitative polymerase chain reaction (qPCR), and electron microscopy.

A key consideration with making a histological diagnosis is that BKPyVAN is a characteristically focal disease. As such, a core obtained outside infected foci may be misleading. Additionally, advanced BKPyVAN may be associated with extensive tubular atrophy and interstitial fibrosis, resulting in a lack of cells demonstrating typical cytopathic changes. Discordant findings between multiple cores have been reported to occur in 36.5% of cases [12]. As such, sampling two biopsy cores, including portions of medulla in at least one core, is required to make a comprehensive histological assessment [13]. While this practice increases the likelihood of correctly diagnosing or excluding BKPyVAN, false negatives remain a possibility, and the full clinical picture should always be taken into account. Multiple classification systems have been suggested to enable stratification of BKPyVAN and to promote inter- and intra-observer consistency [12, 13]. Most recently, the Banff Working Group on Polyomavirus Nephropathy proposed and validated a three-tier system incorporating the degree of intrarenal viral load and interstitial fibrosis, as these two factors accounted for most of the variation in graft function and graft failure at 24 months [13, 14]. However, correlation between this, and other, BKPyVAN classification systems and graft outcomes has not been consistently demonstrated in other historical cohorts [15].

**Figure 1:**
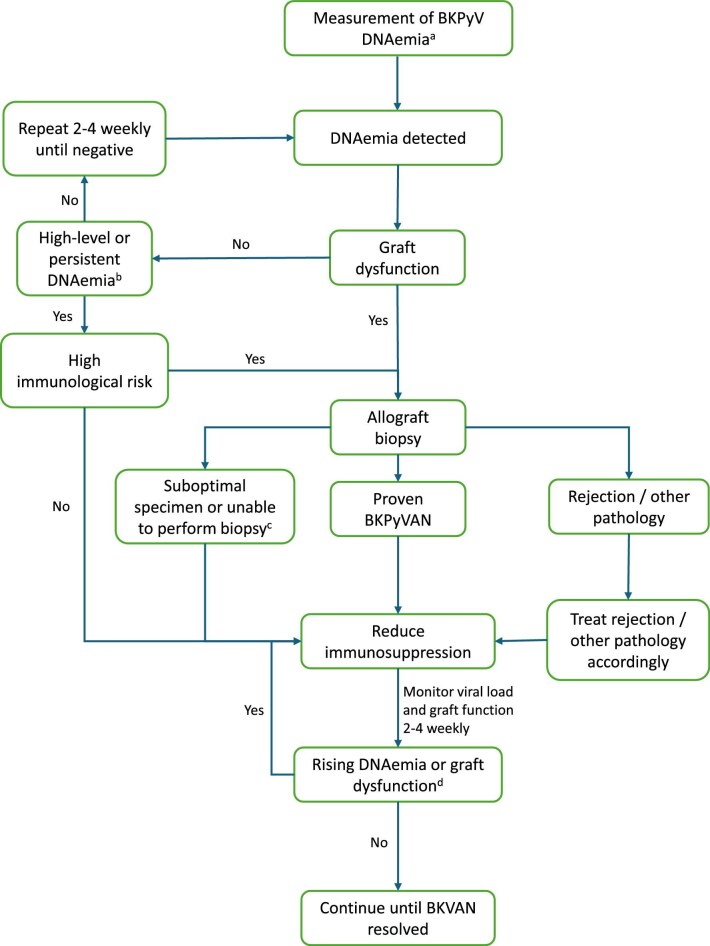
Diagnosis and treatment algorithm for BKPyV in kidney transplant patients. The treatment recommendations should not be applied in cases of concurrent BKPyVAN and acute rejection, where expert opinion and individualized clinical decision making is required. ^a^Either as part of routine screening or as clinically indicated. ^b^High-level DNAemia may be defined as 10^4^ copies/ml, notwithstanding issues with inter-assay variability already discussed. ^c^Assumes no other likely alternative explanation for graft dysfunction. If another explanation is likely, this should be addressed first prior to immunosuppression reduction. ^d^Consider (re)biopsy to rule out concurrent rejection or other pathology.

The challenges associated with biopsy diagnosis has spurred over two decades of work to identify non-invasive diagnostic tools for BKPyV replication and BKPyVAN. Assessment of urinary decoy cells and/or viral DNA by qPCR in urine are generally not recommended due to cost and lack of specificity for BKPyVAN [16, 17], although some have advocated use in identifying patients at higher risk of BKPyVAN early post-transplantation [13, 18]. It is also notable that asymptomatic urinary shedding is relatively common even among non-immunocompromised individuals, exceeding 30% in those >50 years old [5]. Measurement of BKPyV DNAemia by qPCR outperforms diagnostic methods using urine and is established in screening and for-cause detection of viral replication (Fig. 1). The quantity of DNAemia is correlated with the likelihood of BKPyVAN, as well as subsequent risk of interstitial fibrosis and tubular atrophy [7]. A threshold of 10^4^ copies/ml has >88% specificity and up to 50% positive predictive value for BKPyVAN [6, 19], and has been widely adopted in clinical guidelines to define presumptive BKPyVAN and prompt immunosuppression reduction in the absence of histology [20–22]. Although useful to help guide decision making, for reasons discussed next, defining a specific DNAemia threshold to trigger intervention is not without limitations [23]. Sustained DNAemia at lower thresholds may also warrant intervention, particularly in KTRs with new graft dysfunction. There is considerable variability between centres with regards to intervals and duration of DNAemia screening, however, it is accepted that greater frequency screening is warranted in the early post-transplant period when the risk of BKPyV DNAemia is greatest. KDIGO guidelines support monthly screening for the first 3–6 months after transplantation, then 3-monthly until 1 year post-transplant [20]. More recent American Society of Transplantation [21] and Transplantation Society [22] guidelines advocate monthly screening for 9 months, and then 3-monthly up to 2 years post-transplant.

The lack of standardization in BKPyV DNAemia measurement between assays and laboratories hampers interpretation and limits harmonization of data collected across different sites. Uniform adoption of the WHO International Standard and reporting in IU/ml would address this and should be implemented [24], although overquantification of DNAemia when using assays solely targeting the early viral gene region (EVGR) remains a concern due to the WHO standard containing viral subpopulations with large EVGR deletions [25]. Another issue arises from many assays using a genotype I strain as a reference sequence. These assays may be less sensitive for other genotypes, and therefore established diagnostic thresholds for DNAemia may not be met in disease caused by rarer genotypes [26]. These challenges explain the lack of a specified BKPyV DNAemia threshold in the consensus definition of ‘probable’ BKPyAN [23], and highlight the need for standardized assays as a prerequisite of clinical trials in this field. However, notwithstanding these issues and the need for validation in other cohorts, temporal monitoring of BKPyV DNAemia may also support non-invasive prognostication, with evidence that that each log unit decrease in viral load is associated with ∼22% reduced risk of graft loss [27]. Novel approaches for supporting the diagnosis and monitoring of BKPyVAN may include the BKPyV-produced microRNA bkv-miR-B1-5p [28] and also donor-derived cell-free DNA [29], a non-specific marker of allograft injury that could potentially have utility as part of an integrative assessment of intragraft immunopathology. Torquetenovirus replication has been appraised as a surrogate marker of functional immunity and, although predictive of allograft rejection, appears not to be correlated with BKPyV replication [30, 31].

Box 1: Definitions of BKPyV disease in kidney transplant patients* (adapted from Imlay *et al.* 2022 [23])

BKPyV Infection: detection of viral components or intact virions in bodily fluids or tissue specimens. Antibodies reflect prior exposure to BKPyV but the presence of these does not indicate active infection.

BKPyV DNAemia: detection of BKPyV DNA in plasma, serum, or whole blood.

BKPyV Viraemia: often used synonymously with BKPyV DNAemia, although for other viral infections (e.g. CMV) ‘viraemia’ refers to the presence of intact virions determined by viral culture, which is technically challenging for BKPyV.

Proven BKPyVAN: demonstration of active BKPyV replication in renal tissue and concomitant BKPyV DNAemia (histological features of BKPyVAN in the absence of BKPyV DNAemia should prompt consideration of JC polyomavirus nephropathy).

Probable BKPyVAN: must fulfil all the following criteria:

- Biopsy not performed or suboptimal specimen obtained.
- BKPyV DNAemia present (measurement must be repeated).
- Evidence of renal allograft dysfunction with no likely alternative explanation.

*Alternative consensus and society definitions of BKPyV disease such as presumptive BKPyVAN (BKPyV DNAemia >10^4^ copies/ml)[22] exist.

## TREATMENT STANDARDS

Immunosuppression reduction is the mainstay of management of BKPyVAN [32]. However, comparison of available observational evidence is challenging due to variations in induction and maintenance immunosuppression regimens, immunosuppression reduction strategies, and target drug levels, as well as definitions of BKPyVAN and of viral clearance. Historical studies have reported resolution of DNAemia in significant proportions of patients after reduction in immunosuppression [33–36], but these are difficult to definitively interpret due to variations in immunosuppression practice/eras and drug levels, and inclusion of patients with lower levels of BKPyV DNAemia than are correlated with BKPyVAN (e.g. most patients having BKPyV DNAemia levels of <10^4^ copies/ml [34, 35]). One study that did stratify participants by degree of DNAemia found an improvement in BKPyV DNAemia levels with immunosuppression reduction, but without reporting a statistical difference in immunosuppression levels versus other groups [36]. The reduction in DNAemia occurred at an average of 200 days post-immunosuppression reduction, when other effects such as time since induction immunosuppression might be confounding factors. On top of this, 14% of those receiving immunosuppression reduction subsequently developed acute cellular rejection. While other studies specifically considering immunosuppression reduction have not reported similar rates [33–35], this may be because the patients included were more highly immunosuppressed at baseline e.g. receiving triple immunosuppression. Several studies have reported no impact of immunosuppression reduction on graft survival, but such conclusions are hampered by short follow-up periods [34, 36]. Even when longer follow up is available [33–35, 37], it remains short enough (≤5 years) that it cannot be relied on to predict trends over lengths relevant to graft half-lives that exceed 10 years in the modern era [38]. Time-to-clearance after immunosuppression reduction is prolonged in biopsy-proven BKPyVAN [33] and DNAemia may recur, necessitating further immunosuppression reduction. Further complexity arises in patients with concurrent BKPyVAN and rejection: a well-recognized phenomenon [39]. Additionally, reduction in immunosuppression may lead to sensitization without overt rejection, contributing to later graft loss and presenting a challenge for re-transplantation.

These issues are even more important when considering the increasing proportion of patients transplanted across antibody barriers in whom the reduction of immunosuppression is a higher risk manoeuvre, and so potentially less likely to be undertaken. Even when deemed clinically appropriate in this group, such a reduction could reasonably be expected to embed a negative long-term impact on graft function. Caution is also required in patients who are recipients of another transplanted organ. Given all these issues, while undertaking careful immunosuppression reduction remains the mainstay of BKPyVAN management, direct-acting therapies are clearly needed for the most effective management of this patient group.

### Evidence about which immunosuppressants to alter in BKPyVAN

Observational studies have reported success with the initial reduction of either antiproliferatives or calcineurin inhibitors. Further stepwise decreases of immunosuppressive burden may be required for patients with high or sustained BKPyV DNAemia. There are several ways this can be performed. Two of the more common strategies [21] involve:

- Reduction of calcineurin inhibitor target trough level by 25%–50%, followed by reduction of antiproliferative by 50%, followed by cessation of antiproliferative
- Reduction of antiproliferative by 50%, followed by reduction of calcineurin inhibitor target trough by 25%–50%, followed by cessation of antiproliferative

Evidence exists in support of either approach.

#### Reducing calcineurin inhibitors first

In a prospective study of 644 consecutive KTRs, of whom 105 developed BKPyV DNAemia, a CNI-first reduction strategy resulted in viral clearance in 96% of patients [40], although it should be noted that 23% of DNAemic participants had <10^4^ copies/ml, and most were receiving triple immunosuppression with tacrolimus, MMF, and steroids as well as having received induction therapy. KTRs with DNAemia greater than 10^4^ copies/ml or proven BKPyVAN required more extensive reduction of immunosuppression, and a rejection rate of 25% was seen in all study arms. Overall graft outcomes at a median 6.6 years of follow up were comparable to KTRs without DNAemia, although median MDRD eGFR was 10 ml/min less in the BKPyVAN group at viral clearance (reaching statistical significance). This could be considered an earlier outcome measure than graft/patient survival, differences in which require longer follow up to observe. Interestingly, the study cohort contained a significant proportion of transplants across HLA and ABO barriers, with donor-specific antibodies present in 100/644 (17%) of those undergoing screening, and 60/644 (9%) ABO incompatible. Longer-term follow up of this higher risk population would be of great interest.

#### Reducing antiproliferatives first

Initial discontinuation of antimetabolites (mycophenolate or azathioprine) among 23 patients after identification of BKPyV DNAemia by screening, followed by CNI reduction in those with sustained DNAemia, resulted in 95% achieving viral clearance and comparable allograft outcomes at 5 years to those without DNAemia, notwithstanding the limitations noted before [34, 37].

#### Other strategies

Evidence is lacking for the simultaneous reduction of both agents, but this may be considered in patients with lower immunological risk and significant graft dysfunction due to BKPyVAN. Some clinicians advocate complete removal of the anti-proliferative and the addition of steroid to a CNI based regimen, only reducing CNI trough levels if this is insufficient to resolve the BKPyVAN/DNAemia, although without clear evidence base.

#### No robust evidence supports switching to alternative calcineurin inhibitors or changing to mTOR inhibitors

Compared to ciclosporin, tacrolimus has inconsistently been associated with greater risk of developing BKPyVAN [41], with *in vitro* studies suggesting it may support BKPyV replication in RTE cells [42]. Conversely, ciclosporin may suppress viral infection [43]. Additionally, ciclosporin inhibits the enterohepatic circulation of the major mycophenolic acid metabolite, MPA glucuronide, resulting in lower immunosuppressive effect compared to mycophenolate in combination with tacrolimus [44]. A prospective study of 24 KTRs with proven BKPyVAN receiving triple immunosuppression reported viral clearance within a mean 2.7 months after switching from tacrolimus to low-dose ciclosporin and 100% graft survival [45]. Interpretation of these findings is severely limited by the lack of a control group, and requires validation. Furthermore, although tacrolimus was elsewhere associated with higher incidence of BKPyV viruria than ciclosporin [34], graft function at 5 years among the cohort was greater among those receiving tacrolimus (eGFR 63 vs 52 ml/min, *P* = .001)[37].

A registry study identified that mTOR inhibitor use was associated with lower incidence of BKPyVAN [41], although it is unclear whether this was as baseline immunosuppression or following conversion. *In vitro*, sirolimus appears to limit viral replication [42] and, unlike tacrolimus, does not impair functionality of BKPyV-specific T cells [46]. A meta-analysis of RCTs evaluating mTOR inhibitor-containing immunosuppression regimens indicated a trend towards decreased incidence of BKPyV DNAemia [47]. However, the quality of evidence was low. These observations underpinned the recent BKEVER study, a multicentre RCT comparing switching from MMF to everolimus versus reduction of MMF dose in 130 KTRs with BKPyV DNAemia, where both groups also had the CNI dose reduced [48]. Here, switching MMF to everolimus (plus CNI reduction) resulted in fewer patients achieving the primary endpoint of BKPyV DNAemia clearance at 6 months versus MMF and CNI reduction alone (55.7% vs 81.3%; odds ratio 3.4; *P* = .003), suggesting no role for mTOR inhibitors in the management of BKPyV disease.

### Adjunctive therapies

No drug treatment can be recommended in isolation due to lack of robust efficacy data. There have been two RCTs assessing fluoroquinolone antibiotics for BKPyV infection post-transplantation. Prophylaxis with either levofloxacin [49] or ciprofloxacin [50] showed no benefit in terms of BKPyV viruria, DNAemia and/or BKPyVAN, and some evidence of harm in the form of increased fluoroquinolone-resistant bacterial infections [50]. No RCTs assessing other adjunctive therapies have been reported to date.

Beyond fluoroquinolones, adjunctive agents that have been variably administered and studied both *in vitro* and in small clinical studies include leflunomide, intravenous immunoglobulin (IVIG) and cidofovir (Table 1). A meta-analysis failed to identify benefit of any of these, either alone or in combination [32]. Only IVIG has an (inter)national clinical guideline recommendation for use as an adjuvant in KTRs with insufficient response to reduced immunosuppression or as adjuvant to reduced immunosuppression to prevent acute rejection in patients with high immunological risk [22]. This recommendation is based as much on the relative safety and tolerability of IVIG as an adjunctive treatment [51], as it is on the limited evidence from single-centre observational studies suggesting improved viral clearance versus immunosuppression reduction alone [51, 52].

**Table 1:** Adjunctive therapies dose, precautions, toxicities, highest level of evidence, and guideline recommendations for use.

Drug	Example dosing regimen	Toxicities	Precautions and monitoring	Guideline supported^a^	Other considerations
Intravenous Immunoglobulin	300 mg/kg 3-weekly Note existing studies have used variable dosing regimens	Infusion reactions Anaphylaxis	IgG levels may be used to titrate dose	Yes [22]^b^ Equivocal [21]	A multicentre RCT assessing a human monoclonal VP1-specific IgG1 is ongoing (ClinicalTrials.gov identifier: NCT04294472)
Leflunomide	100 mg loading, then 40 mg daily	Bone marrow suppression Hepatotoxicity Haemolysis	Teratogenic with relevance to both males and females^c^ Monitor FBC and LFT fortnightly for 6 months, then 8-weekly	No [22] Equivocal [20, 21]	Variable inter-patient metabolism
Fluoroquinolones	500 mg daily levofloxacin	Achilles tendonitis Gastrointestinal upset Rash	History of tendon damage related to quinolones	No [22] Equivocal [21]	Lack of efficacy demonstrated in two RCTs [49, 50]
Cidofovir	0.25–1 mg/kg at 1–3-week intervals. Increase dose depending on response and toxicity	Nephrotoxicity Anterior uveitis and other eye manifestations	Monitor renal function, proteinuria, and FBC at least 48 hours prior to each dose Regular eye examination e.g. fortnightly during treatment	No [22] Equivocal [21]	Consider concomitant administration with oral probenecid Avoid concomitant use with tenofovir due to increased risk of Fanconi syndrome

a ‘Yes’ indicates published clinical guideline states recommendation for use in specific circumstances after, or in concert with, immunosuppression reduction. ‘No’ indicates specific recommendation against use. ‘Equivocal’ indicates no specific recommendation for/against use.

b Suggested as adjuvant in KTRs with insufficient response to reduced immunosuppression or as adjuvant to reduced immunosuppression to prevent acute rejection in patients with high immunological risk (weak recommendation, grade D).

c Implications for foetal development when given to either men or women who might parent a child in future. Effective contraception should be in use. A 2-year delay is needed before pregnancy on stopping leflunomide (though a washout procedure with cholestyramine or activated charcoal can be used to reduce this timeframe). Men who father a child while taking leflunomide also risk foetal toxicity (congenital abnormalities), although washout period is shorter (3 months).

Further information detailing specific studies examining different treatment strategies can be found in supplemental tables S8–S11 in the recent Transplantation Society Consensus Guidelines on the management of BKPyV in KTRs [22].

Overall, as concluded in a recent Cochrane review of interventions for BKPyV disease in KTRs, there is insufficient evidence to support any specific intervention other than immunosuppression reduction [53]. Given the current level of data available, initiation of any adjunctive therapy should be undertaken following maximal acceptable reduction in immunosuppression, and with careful consideration of potential toxicities. Fluoroquinolones should not be used given RCT evidence demonstrating lack of efficacy, and cidofovir avoided due to significant risk of renal and ophthalmic toxicity without clear evidence of benefit. Carefully designed and appropriately controlled clinical trials incorporating mechanistic studies, and which evaluate standardized immunosuppression reduction strategies, with and without adjunctive therapies, are needed.

### Re-escalation of immunosuppression after viral clearance

Evidence regarding when/if immunosuppression should be re-escalated following viral clearance is lacking. A single-centre retrospective study of 644 consecutive KTRs reported a rate of BKPyV relapse after DNAemia clearance of 12/101 (12%) [40]. Among these, four cases (33%) occurred in the context of increased immunosuppression for rejection treatment. However, there is evidence that biopsy-proven acute rejection (BPAR) occurs more frequently unless immunosuppression is re-escalated. Another single-centre retrospective study of 88 patients who had immunosuppression reduction after developing BKPyV DNAemia reported that BPAR was less frequent among those who had their immunosuppression subsequently increased [5/44 (11%) versus 14/44 (32%) among those who had no increase in immunosuppression], although BKPyV DNAemia recurred in 10/44 (23%) of those who had immunosuppression re-escalation [54]. A pragmatic approach may be to stratify patients according to their immunological risk. Following resolution of DNAemia, for low immunological risk patients who have had no adverse consequences following reduction in intensity of immunosuppression, it may be appropriate to continue this in the longer term. Conversely, in higher immunological risk patients, gradual augmentation of immunosuppression can be undertaken, together with regular screening for recurrence of BKPyV DNAemia. Further studies evaluating the timing, extent, and nature of re-escalation of immunosuppression after viral clearance are needed, both with and without the context of rejection.

### Re-transplantation after graft loss due to BKPyVAN

The proportion of patients who experience graft loss directly due to BKPyVAN is <2% [9, 10], although history of BKPyVAN does negatively affect long-term graft survival [10]. Evidence regarding the best approach to re-transplantation after graft loss due to BKPyVAN is lacking. Observational data suggest superior graft survival after re-transplantation in patients who lost their first graft due to BKPyVAN compared to those whose transplant failed from other causes [55]. A retrospective study of 31 patients in six US centres indicated clearance of BKPyV DNAemia prior to re-transplantation was associated with absence of DNAemia afterwards and is therefore desirable [56]. However, given the absence of data and the known adverse outcomes associated with prolonged dialysis, persistent BKPyV DNAemia should not be regarded as an absolute contraindication to re-transplantation. Allograft nephrectomy has been shown to rapidly clear BKPyV DNAemia and has been undertaken in patients with persistent DNAemia prior to successful re-transplantation [57]. However, allograft nephrectomy carries the potential for sensitization [58] in addition to surgical complications, and there is no compelling evidence that this mitigates the risk of BKPyV manifesting following the subsequent kidney transplant.

## NEW DEVELOPMENTS IN PATHOPHYSIOLOGY AND OUTCOME PREDICTION

**Figure 2:**
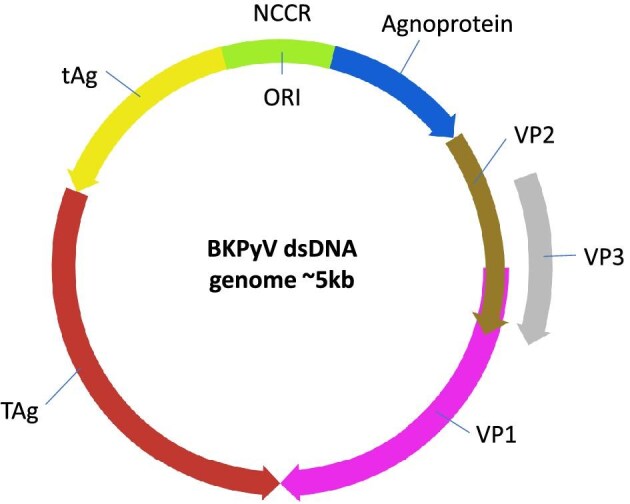
Schematic of dsDNA BKPyV genome. The origin of replication (ORI) is in the NCCR. Open reading frames are represented by arrows. Both the small t antigen (tAg) and the large T antigen (TAg) can undergo alternative splicing (not illustrated). Viral capsid proteins are VP1, VP2, and VP3.

Insights into the fundamental biology of BKPyV infection offer potential avenues to develop targeted treatments and diagnostic tools [59]. Briefly, the BKPyV genome (Fig. 2) has three components: (i) the regulatory non-coding control region (NCCR); (ii) early viral genes (small and large T antigens); and (iii) late viral genes (capsid proteins VP1, VP2, VP3, and agnoprotein). NCCR mutations that affect cell tropism and rates of replication are seen in KTRs with clinically significant BKPyV infection. Greater understanding and identification of these NCCR rearrangements may help identify those at greatest risk of developing BKPyVAN. VP1 binds to cell surface ganglioside receptor GT1b; VP2 and VP3 appear to act as nuclear localization signals. TAg regulates viral gene expression, targeting host tumour suppressor proteins such as p53 and retinoblastoma protein, and facilitates the assembly of a replication complex. After DNA replication, the capsid is formed from VP1 molecules in pentamers, with each VP1 pentamer linking to VP2/3. Newly synthesized genomes are then packaged in virions within the nucleus. Progeny virion release is thought to utilize a cell lytic mechanism, although some evidence suggests an additional role for active secretion [60]. Interestingly, inhibition of the cystic fibrosis transmembrane receptor with glibenclamide prevents infection *in vitro* by disrupting early BKPyV trafficking to the endoplasmic reticulum [61], and virion release appears to be dependent on the agnoprotein, presenting a potential drug target [62]. Further studies unpicking the molecular mechanisms of BKPyV infection are expected to support the development of much-needed therapeutics.

Akin to the risk of cytomegalovirus infection following transplantation, a BKPyV seronegative individual receiving an allograft from a seropositive donor may be at increased risk of BKPyV DNAemia [63]. Pre-existing humoral immunity does not appear to confer protection against BKPyVAN [64], and donor/recipient BKPyV serotype mismatch correlates with risk of DNAemia [65]. Dysfunctional cellular immunity appears to be important in the pathophysiology of BKPyVAN. Multiple studies have identified a lack of peripheral BKPyV-specific IFNγ-producing T cells in KTRs who develop BKPyVAN, and their subsequent emergence following immunosuppression reduction [66, 67]. The detection of these responses temporally correlates with control of DNAemia. These observations may be harnessed as tools to guide tailored immunosuppression reduction and prognostication. A prospective study of 32 paediatric KTRs observed a negative correlation between the frequency of IFNγ-producing BKPyV-specific CD4^+^ and CD8^+^ T cells at, or soon after, diagnosis and the subsequent duration of DNAemia [68]. These authors proposed thresholds of ≥0.5 cells/µl for BKPyV-specific CD4^+^ T cells and/or ≥0.1 cells/µl for BKPyV-specific CD8^+^ T cells predicted transient, self-limited DNAemia not requiring immunosuppression reduction (positive predictive value 1; negative predictive value 0.86). These cut-offs require validation in larger cohorts with varying immunosuppression regimens, and ultimately RCTs to determine whether using T cell monitoring to guide management leads to improved graft outcomes.

Insights from studies of peripheral immune responses are limited in that the immunopathology of BKPyVAN occurs within the graft. Molecular approaches, most notably the Molecular Microscope Diagnostic System [69], have been used to phenotype distinct forms of allograft injury, however, these techniques have to date been unable to adequately discriminate BKPyVAN from rejection [70, 71]. They have, however, shed light on the limitations of histology in distinguishing tubulitis and interstitial infiltrate mediated by TCMR, in the presence of BKPyV [72]. Novel bioinformatics approaches including single-cell and spatial transcriptomics/multiomics may provide greater insight into the intragraft immune landscape of BKPyVAN and reveal pathways that may be harnessed in tools for diagnosis, prognostication, and development of therapeutics.

## NEW DEVELOPMENTS IN MANAGEMENT

Harnessing T cell immunity through adoptive immunotherapies may be one approach to treat BKPyV in KTRs. A single case report has described successful viral clearance following *ex vivo* expansion and infusion of allogeneic BKPyV-reactive T cells from that patients’ daughter, although the graft was lost [73]. The authors speculated that advanced fibrosis of the graft at the time of treatment may explain the lack of clinical benefit. Posoleucel, a multivirus-specific T cell therapy, has recently shown promise in control of BKPyV DNAemia among KTRs in a phase 2 study, with no safety concerns [74]. However, phase 3 trials in allogeneic stem cell transplant recipients were recently abandoned for futility at interim analyses. Novel immunomodulatory therapies to enhance T cell activity, for example extracorporeal photopheresis [75], are being evaluated. Other therapies under investigation include a VP1-specific human monoclonal neutralizing antibody (nAb) therapy (MAU868), which is reported to show activity against all BKPyV genotypes and for which promising data has been reported in phase 2 studies, albeit on low numbers (*n* = 28) [76]. Similarly, recombinant BKPyV virus-like particles can elicit robust antibody titres [77] and, given evidence for nAbs pre-transplantation mediating some protection against DNAemia [78], may represent a potential route towards pre-transplant vaccination. Furthermore, one study has suggested that prophylactic administration of IVIG in KTRs with low BKPyV-specific nAb titres is associated with lower incidence of BKPyV DNAemia, providing proof-of-concept for IVIG/nAbs as a preventative therapy [79]. The results from a phase 3 study (NCT 04222023) are awaited. Small molecule drugs targeting BKPyV remain elusive.

## SUMMARY

A definitive diagnosis of BKPyVAN requires histology, the focal nature of which necessitates sampling of multiple tissue cores. Screening of BKPyV DNAemia is widely established, with defined thresholds increasingly used to guide proactive immunosuppression reduction. Interpretation of these results is limited by intra- and inter-laboratory variability in diagnostic assays. Uniform adoption of the WHO International Standard and reporting in IU/ml will support harmonization of data across centres and is a prerequisite for any future clinical trials to evaluate immunosuppression reduction strategies and novel therapies. Immunosuppression reduction is the mainstay of management, and stepwise reductions in the immunosuppressive burden may be required to achieve viral clearance, balanced against the individual patients’ immunological risk. Adjunctive therapies, particularly IVIG, may be considered only after maximum acceptable reduction in immunosuppressive burden. Efforts to elucidate the fundamental biology of BKPyV infection and characterize the key immunological correlates of protection offer routes towards the development of much-needed targeted therapeutics and prognostic tools.

## Data Availability

No new data were generated or analysed in support of this research.
